# Efficacy of SPG-ODN 1826 Nanovehicles in Inducing M1 Phenotype through TLR-9 Activation in Murine Alveolar J774A.1 Cells: Plausible Nano-Immunotherapy for Lung Carcinoma

**DOI:** 10.3390/ijms22136833

**Published:** 2021-06-25

**Authors:** Mohammed F. Aldawsari, Ahmed Alalaiwe, El-Sayed Khafagy, Ahmed Al Saqr, Saad M. Alshahrani, Bader B. Alsulays, Sultan Alshehri, Amr S. Abu Lila, Syed Mohd Danish Rizvi, Wael A. H. Hegazy

**Affiliations:** 1 Department of Pharmaceutics, College of Pharmacy, Prince Sattam Bin Abdulaziz University, Al-kharj 11942, Saudi Arabia; moh.aldawsari@psau.edu.sa (M.F.A.); a.alalaiwe@psau.edu.sa (A.A.); a.alsaqr@psau.edu.sa (A.A.S.); sm.alshahrani@psau.edu.sa (S.M.A.); b.alsulays@psau.edu.sa (B.B.A.); 2 Department of Pharmaceutics and Industrial Pharmacy, Faculty of Pharmacy, Suez Canal University, Ismailia 41522, Egypt; 3 Department of Pharmaceutics, College of Pharmacy, King Saud University, Riyadh 11451, Saudi Arabia; salshehri1@ksu.edu.sa; 4 Department of Pharmaceutics and Industrial Pharmacy, Faculty of Pharmacy, Zagazig University, Zagazig 44519, Egypt; a.abulila@uoh.edu.sa; 5 Department of Pharmaceutics, College of Pharmacy, University of Hail, Hail 81442, Saudi Arabia; sm.danish@uoh.edu.sa; 6 Department of Microbiology and Immunology, Faculty of Pharmacy, Zagazig University, Zagazig 44519, Egypt; waelmhegazy@daad-alumni.de

**Keywords:** schizophyllan, CpG ODN 1826, inflammatory cytokines, Th1, lung cancer, M1 and M2

## Abstract

Alveolar macrophages are the first line of defense against intruding pathogens and play a critical role in cancer immunology. The Toll-like receptor (TLR) family mediates an important role in recognizing and mounting an immune response against intruding microbes. TLR-9 is a member of the intracellular TLR family, which recognizes unmethylated CG motifs from the prokaryotic genome. Upon its activation, TLR-9 triggers downstream of the MyD-88-dependent transcriptional activation of NF-κB, and subsequently results in abundant inflammatory cytokines expression that induces a profound inflammatory milieu. The present exploratory investigation aimed at elucidating the potency of schizophyllan for entrapping ODN 1826 (SPG-ODN 1826)-mediated stimulation of TLR-9 in provoking an inflammatory-type response in murine alveolar macrophages. Schizophyllan (SPG), a representative of the β-glucan family, was used in the present study as a nanovehicle for endosomal trafficking of CpG ODN 1826. TEM analysis of SPG-ODN 1826 nanovehicles revealed that the prepared nanovehicles are spherical and have an average size of about 100 nm. Interestingly, SPG-ODN 1826 nanovehicles were competent in delivering their therapeutic payload within endosomes of murine alveolar macrophage (J774A.1) cells. Exposure of these nanovehicles within LPS stimulated J774A.1, resulted in a significant provocation of reactive oxygen species (ROS) (*p* < 0.01) in comparison to CpG ODN 1826 alone. Moreover, the formulated nanovehicles succeeded in generating a profound Th1-based cytokine profile constituted by enhanced expression of IFN-γ (*p* < 0.001) and IL-1β (*p* < 0.001) inflammatory cytokines. These findings clearly indicated the immunostimulatory potential of SPG-ODN 1826 nanovehicles for inducing the Th1-type phenotype, which would certainly assist in skewing M2 phenotype into the much-desired M1 type during lung cancer.

## 1. Introduction

Macrophages (Mϕs), derived from the monocyte lineage, are a prerequisite for neutralizing the non-self constituents of the human body as well as the apoptotic or damaged cells during homeostasis [1]. Mϕs play another important role in shaping the adaptive immune response within individual post recognition of intrinsic or extrinsic stress [2]. Tissue-resident Mϕs are well-distributed within different organs including lungs, where they mediate the critical function of antigen presentation and thus promote adaptive immunity during different clinical manifestations [3]. Antigen-presenting cells (APCs) such as Mϕs and dendritic cells (DCs) recognize the non-self moieties due to the presence of pathogen associated molecular patterns (PAMPs) on the invading microbes. Furthermore, antigenic moieties are perceived by pathogen recognition receptors (PRRs), such as Dectin-1 (a major β-glucan receptor), mannose receptors, and others expressed primarily on APCs [4]. 

The PRRs super-family of innate immune receptors is constituted by an important family of Toll-like receptors (TLRs), whose 10 functional members have been characterized in humans. TLRs are transmembrane type-I proteins expressed both extracellularly and intracellularly. The functional expression of different TLRs such as TLR-2, TLR-4, and TLR-9 is documented at mRNA levels in human alveolar macrophages [5,6]. TLR-9, expressed intracellularly within endosomes, is a member of an intracellular TLR family that is responsible for recognizing prokaryotic or unmethylated CG motifs from the microbial genome. The binding of CG-rich regions of prokaryotic DNA with TLR-9 triggers downstream transcriptional activation of NF-κB through the MyD88-dependent pathway, and eventually culminates in expressing inflammatory cytokines such as IFN-γ, IL-1β, and IL-2 [7,8]. Importantly, TLR-9 agonists efficiently mediate inflammatory processes that lead to the enhancement in the uptake and killing of tumor cells [9,10]. Moreover, TLR-9 agonists were employed as potent adjuvants of cancer vaccines against various types of cancers [9].

Lung cancer is a serious concern and represents the foremost cause of cancer-related mortality globally [11,12]. Lung cancer is clinically differentiated into small-cell and non-small-cell lung cancer (NSCLC), where the latter is responsible for 85% of total lung cancer cases reported [13,14]. Tumor-associated macrophages (TAMs), existing in the cancer microenvironment, are the key cells that affect the formation, growth, and metastasis of cancers by interacting with cancer cells [15]. Despite extensive research conducted during the last decades, it is still unclear whether Mϕs can exhibit different polarized states during cancer progression. With different stimuli, macrophages can be polarized into M1 macrophages (pro-inflammatory or classically activated Mϕs) or M2 macrophages (anti-inflammatory or alternatively activated Mϕs) [16]. M1 Mϕs have been acknowledged for mounting an effective immune response toward microbial infections and cancer [17]. By contrast, M2 Mϕs are considered the key cells that create an immunosuppressive tumor microenvironment (TME) by producing growth factors, cytokines, chemokines, and/or eliciting the release of inhibitory immune checkpoint proteins in T cells [18]. Recently, it was demonstrated that profound levels of TAMs (M2 type) in lung cancer correlate with poor prognosis and survival rate of patients due to the augmented expression of vascular endothelial growth factors (VEGF), especially VEGF-A and VEGF-C [19,20]. Although skewing the axis of these polarized states depends substantially on various intrinsic cues, shifting toward M1 Mϕs stimulation can enhance an effective immune response to cancer [16,21]. Numerous TLR agonists were used to reprogram M2 Mϕs into M1 type, which may be important in cancer immunotherapy [22]. Among these agonists, CpG ODN is a TLR-9 agonist that was employed as mono or combined immunotherapy with conventional chemotherapy and other therapies in multiple human clinical trials, phases II and III, against several types of cancer [9,22,23,24]. As such, we aimed to screen the ability of some molecules to activate TLR-9 that can stimulate M1 Mϕs to exert an efficient anticancer immune response. 

Schizophyllan or SPG is a constituent of the β-glucan family isolated from *Schizophyllum commune* and is recognized by Dectin-1 receptors on Mφ and other APCs [25]. SPG has been explored substantially for its applicability in delivering short sequences of nucleic acids intracellularly within immune cells, namely Mϕs and DCs. SPG-derived nanoparticles were documented to behave as a Dectin-1 non-agonist and deliver the immunostimulatory payload within endosomal TLR-9 [26]. Owing to these observations, we hypothesized that SPG nanovehicles entrapping CpG ODN 1826 could be used to instigate the desired M1 or inflammatory phenotype in murine alveolar Mϕs J774A.1 cells, and could thus represent plausible nano-immunotherapy for lung carcinoma.

## 2. Results

### 2.1. Size, Morphology, and Entrapment of CpG ODN 1826 within SPG-ODN 1826 Nanovehicles 

**Figure 1:**
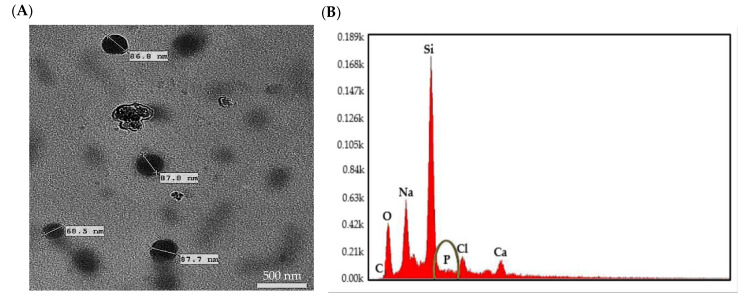
Characterization of the synthesized nanovehicles by (**A**) transmission electron microscopy and (**B**) energy dispersive X-ray. The spectra elucidated the presence of a phosphorous peak, corresponding to the phosphorothioate (PS) backbone of the respective ODN. (Scale bar = 500 nm; magnification = 10,000×).

The TEM micrograph of SPG-ODN 1826 nanovehicles formulated during our study showed near-spherical morphology and were found to be about 100 nm in diameter as evaluated through electron microscopy (Figure 1A). EDX analysis was undertaken to ascertain the entrapment of CpG ODN 1826 with SPG. Figure 1B shows the elemental peaks of the SPG-ODN 1826 nanovehicle. The presence of a phosphorus peak, corresponding to the phosphorothioate (PS) backbone of the respective ODN, confirmed the efficient entrapment of CpG ODN 1826 within the SPG nanovehicle [27]. The EDX spectra show additional peaks corresponding to the different constituents of glass slides used during the analysis. 

### 2.2. SPG-ODN 1826 Nanovehicles Delivered TLR-9 Agonist within Endosomes 

**Figure 2:**
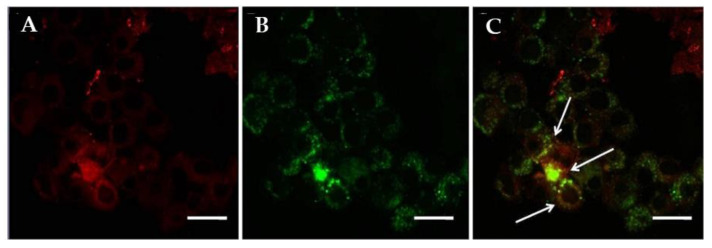
Confocal scanning laser photomicrograph exhibiting the endosomal internalization of SPG-ODN 1826 nanovehicles within J774A.1. (**A**) The plasma membrane is red due to the stain of FM 4-64 whereas (**B**) the internalized SPG-ODN 1826 nanovehicles appear green due to the presence of tagged FAM-CpG ODN 1826. (**C**) Endosomal localization of SPGNPs within alveolar macrophages J774A.1 cells was tracked using yellow fluorescence created by the overlay images of FM4-54 and FAM stain that resulted in a yellow color (indicated by arrows). Magnification = 32×, scale bar = 20 μm.

Owing to its intracellular localization and other associated intracellular factors, delivery of biological payloads to endosomes remains a challenging task. Confocal scanning laser microscopy (CSLM) analysis exhibited the relevance of SPGs in delivering TLR-9 agonist. The results illustrated that the plasma membrane of J774A.1 exhibited red fluorescence due to the affinity of FM4-64 dye toward the hydrophobic domains (Figure 2A). The FAM-associated green fluorescence indicated the intracellular localization of SPG-ODN 1826 nanovehicles (Figure 2B). The overlay image (Figure 2C) of FM4-64 and FAM fluorescence exhibits a yellow color that indicates endosomal localization of SPGNPs within the alveolar macrophages. 

### 2.3. Synthesized Nanovehicles Elucidated Non-Toxic Effects of J774A.1 Cells 

**Figure 3:**
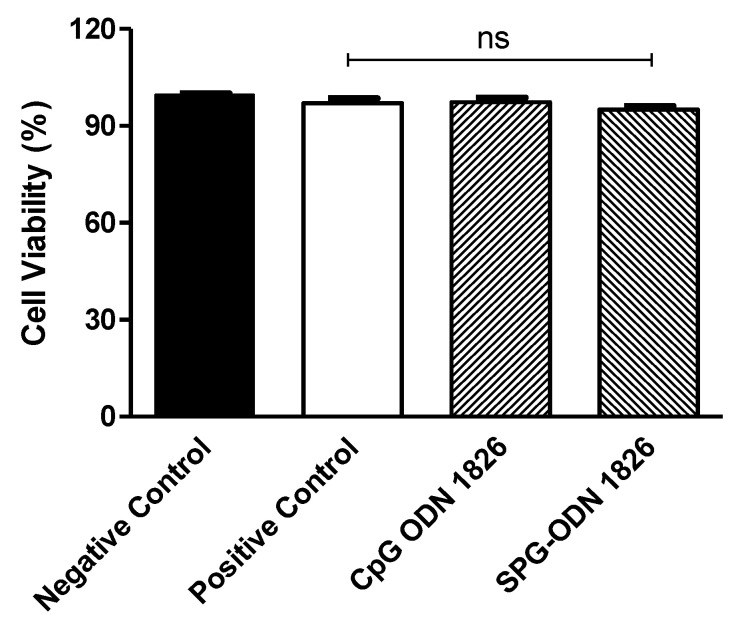
Cell viability of J774A.1 cells treated with different dilutions of synthesized SPG-ODN 1826 nanovehicles for 24 h using an MTT assay. The data presented are the mean ± SEM of three individual experiments, where each was performed in triplicate. Statistical significance between different groups was evaluated using one-way ANOVA where ns (non-significant) indicates *p* > 0.05.

The treatment of J744A.1 cells with SPG-ODN 1826 for 24 h was used to ascertain any plausible cytotoxicity on these cells. The results of the MTT assay clearly outlined that the formulated nanoformulation at different dilutions did not exert any potential cytotoxic effects on J774A.1 cells (Figure 3).

### 2.4. SPG-ODN 1826 Augmented ROS Levels within J774A.1 Cells 

**Figure 4:**
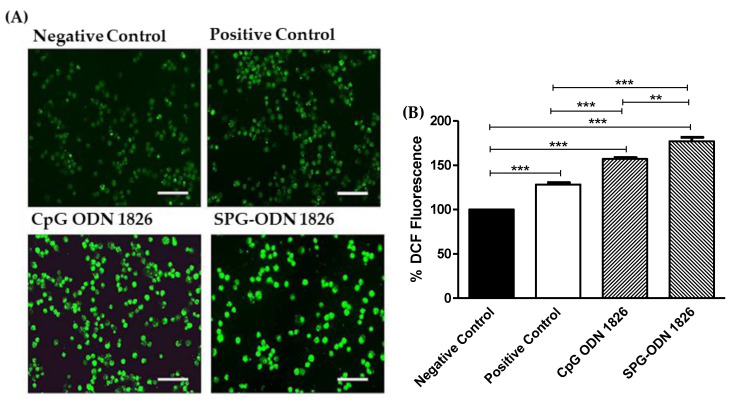
Augmentation of ROS levels within J774A.1 cells treated with TLR-9 agonist and SPG-ODN 1826 nanovehicles. (**A**) Photomicrographs indicating augmented ROS in DCF-DA-positive J774A.1 cells treated with CpG ODN 1826 and nanovehicles. (**B**) Quantification of DCF-DA-mediated fluorescence within J774A.1 cells treated with TLR-9 agonist and formulated nanovehicles. Scale bar = 100 µm. Data presented are the mean ± SEM of three individual experiments, where each was performed individually thrice. Statistical significance between different groups was ascertained using one-way ANOVA and Tukey’s post hoc test where ** *p* < 0.01 and *** *p* < 0.001.

DCF-DA-mediated fluorescence was used to qualitatively and quantitatively assess the alterations within ROS levels induced by SPG-ODN 1826 in comparison to CpG ODN 1826. The photomicrographs indicated increased DCF-DA-mediated fluorescence in J774A.1 cells exposed to CpG ODN 1826, which was further increased in SPG-ODN 1826 nanovehicles-exposed cells (Figure 4A). During the quantitative estimation of ROS, it was evident that with post-stimulation with LPS, the ROS level significantly increased within J774A.1 cells (positive control, *p* < 0.001) in comparison to the negative control. Treatment with SPG-ODN 1826 nanovehicles significantly triggered the production of elevated levels of ROS (177 ± 4.35%; *p* < 0.01) compared to cells treated with CpG ODN 1826 (157 ± 1.52%) (Figure 4B).

### 2.5. SPG-ODN 1826 Nanovehicles Induced Synthesis of Inflammatory Cytokines 

**Figure 5:**
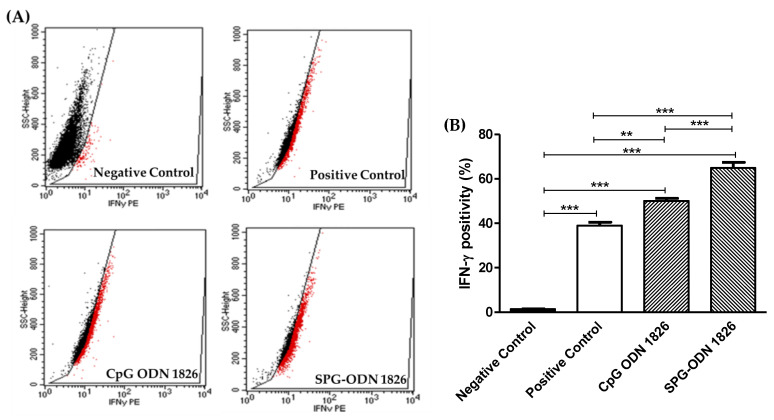
Intracellular staining of IFN-γ within cells. (**A**) PE-mediated positivity of J774A.1 against intracellular levels of IFN-γ as assessed during flow cytometric analysis; Black dots indicate IFN-γ negative cells, while red dots indicate IFN-γ positive cells. (**B**) quantified levels of IFN-γ within J774A.1 cells treated with CpG ODN-1826 and SPG-ODN 1826 nanovehicles with and without LPS stimulation. Data presented are mean ± SEM of three individual experiments, where each was performed individually thrice. Statistical significance between different groups was ascertained using one-way ANOVA and Tukey’s post hoc test where ** *p* < 0.01 and *** *p* < 0.001.

Flow cytometric analysis was conducted to quantify the intracellular levels of signature inflammatory cytokines in J774A.1 cells. The analysis elucidated that there was a substantial escalation of 1.28-fold in the IFN-γ-positive cell population within J774A.1 cells treated with TLR-9 agonist (50.12% ± 1.07%) compared to LPS-stimulated cells (38.97% ± 1.56%; *p* < 0.01). Treatment with formulated nanovehicles further enhanced the IFN-γ- positive population of Mϕs (64.87% ± 2.57%) by slightly more than 1.5-fold in comparison to the LPS-stimulated positive control (*p* < 0.001; Figure 5). 

**Figure 6:**
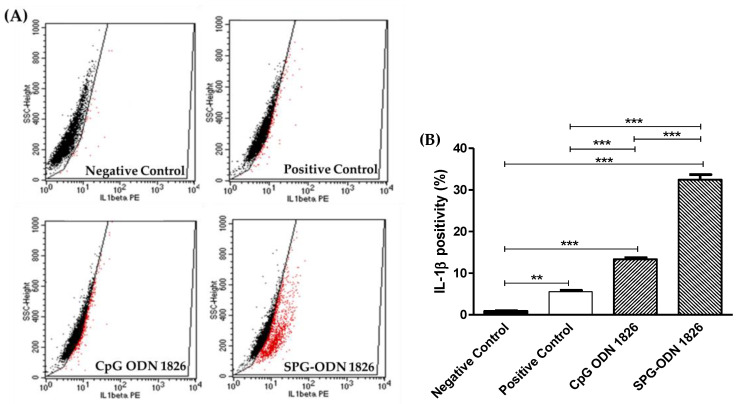
Intracellular staining of IL-1β within cells treated with various regimes formulated during the study. (**A**) PE-mediated positivity of J774A.1 against intracellular levels of IL-1β as assessed during flow cytometric analysis; Black dots indicate IL-1β negative cells, while red dots indicate IL-1β positive cells. (**B**) quantified levels of IFN-γ within J774A.1 cells treated with CpG ODN-1826 and SPG-ODN 1826 nanovehicles with and without LPS stimulation. Data presented are the mean ± SEM of three individual experiments, where each was performed individually thrice. Statistical significance between different groups was ascertained using one-way ANOVA and Tukey’s post hoc test where ** *p* < 0.01 and *** *p* < 0.001.

Assessment of intracellular IL-1β levels (Figure 6) further elucidated the efficacy of the synthesized nanovehicles. The exposure to CpG ODN 1826 resulted in a population increase in IL-1β-positive cells by slightly more than 2.5-fold (13.67% ± 0.19%; *p* < 0.001) in comparison to LPS-stimulated J774A.1 cells (5.55% ± 0.34%). Treatment with nanovehicles (34.26% ± 1.23%) further increased the population of IL-1β-positive cells by ~six-fold in comparison to the positive control cells (*p* < 0.001). As in the case of IFN-γ, SPG-ODN 1826 nanovehicles were competent in enhancing the population of IL-1β-positive Mϕs more significantly than TLR-9 agonist (*p* < 0.001). 

## 3. Discussion

Lung cancer is still the top cause of cancer-related mortality, and its treatment constitutes a major challenge. Regardless of the previous failure of immunotherapy in the treatment of lung cancer, increasing enthusiasm in cancer immunotherapy recently emerged [28]. The implication of nanotechnology has recorded a surge during the last couple of decades owing to the efficiency of nanovehicles in delivering the therapeutic cargo within different anatomical positions in the body [29,30,31]. In the present study, we aimed to explore the potency of schizophyllan, or SPG, as an established member of the β-glucan family and the mediated impelling of TLR-9 for prompting an inflammatory-type response in murine alveolar macrophages.

The β-glucan family comprises a group of β-D-glucose polysaccharides occurring in the bacterial or fungal cell walls [32]. Schizophyllan, or SPG, is an established member of the β-glucan family. Disintegrated β-glucan has been reported as a ligand for natural killer (NK)-like receptors (Dectin-1 receptors). Dectin-1 receptors are involved in the innate immune responses against fungi, but they can also recognize unidentified endogenous ligands on T cells that can consequently induce an efficient cellular response [33,34]. Interestingly, Dectin-1 acts as a major β-glucans receptor on a Mφ and contributes to Mφ-mediated cellular immune response against microbes and cancers [35].

TLR-9 is an endosomal Toll-like receptor, and its expression has been widely reported in various APCs including macrophages [36]. Using synergistic models, it was documented that agonist-mediated activation of TLR-9 either alone or in combinatorial approaches with chemotherapeutic and targeted therapeutic results in amelioration of tumor growth [9]. Furthermore, TLR9 agonists were shown to shift M2 Mϕs to the M1 type, which, in accordance, can enhance an efficient immune response against cancer cells [9,16]. In addition to the finding that Dectin-1 regulates TLR-9-dependent gene expression, Dectin-1-mediated spleen tyrosine kinase (Syk) activation is required for TLR-9 trafficking to β-glucans [37]. In this context, we hypothesized that SPG β-glucan can activate TLR-9 and M1 Mϕs to initiate an effective immune response against lung cancer cells.

Cancerous cells show augmented ROS levels, and treatment of such cells by pro-oxidants has emerged as an effective strategy [38,39]. Furthermore, an increase in intracellular ROS levels is also associated with deflated levels of growth factors, namely VEGF/VEGFR2, which not only results in apoptosis but also causes arrest in cell-cycle transition at the S phase [40]. In this study, the synthesized nanovehicles exhibited their potency in instigating the generation of ROS more significantly in comparison to the TLR-9 agonist, thereby indicating their relevance in stimulating the innate arm of the immune system. Importantly, J774A.1 cells pretreated with N-acetylcysteine (NAC) showed impaired ROS generation even after treatment with either TLR-9 agonist or SPG-ODN 1826 nanovehicles, reaffirming that ROS generation within alveolar Mϕ was augmented by the treatment with TLR-9 agonist and nanovehicles.

Mϕs within lungs, which are confined within the airspaces and are localized within the distal airway under homeostatic or stress conditions, play an indispensable role in inflammation and molding the type of immune response [41,42]. It was reported that alveolar macrophages play a complex and contradictory function in lung cancer. Proinflammatory cytokines secreted by alveolar macrophages were shown to improve anti-tumor functions. However, pro-tumor functions of alveolar macrophages in lung cancer were also indicated [43]. Generally, depending on their environment, Mϕs can adopt numerous functional phenotypes; at least two phenotypes have been designated according to their role in inflammation. Pro-inflammatory M1 Mϕs are induced by Th1 cytokines such as IFN-γ or IL-1β; alternatively, the M2 Mϕs are activated by the Th2 cytokines [44]. In the tumor microenvironment, the M2-polarized Mϕ constitutes the major population of tumor-associated macrophages (TAMs) penetrating the tumor. They promote proliferation and growth of cancer cells along with escalating metastasis and angiogenesis [45]. M2 Mϕs are also competent in altering their phenotypes under the influence of inflammatory cytokines. In this study, the intracellular cytokine staining was conducted by incubating Mϕs with various formulations at different time intervals to prevent their intracellular release from the cells. The intracellular cytokine staining results emphasized that SPG-ODN 1826 nanovehicles could efficiently stimulate TLR-9 as manifested by the profound increase in the positivity of IFN-γ and IL-1β in cells, which, however, was further augmented post-treatment with SPG-ODN 1826 nanovehicles. IFN-γ, a signature inflammatory cytokine, was repeatedly reported to induce the production of IFN-γ inducible protein (IP-10), an efficient anti-tumor molecule involved in attenuating tumor angiogenesis. Moreover, it is also well-known that IFN-γ acts as an important stimulus for skewing the Mϕ phenotype into the M1 type, which exerts its anti-cancer efficacy through immune mediators, specifically iNOS, TNF, and IL-6 [43].

Collectively, the results of our study clearly demonstrated that TLR-9-agonist-mediated immunostimulatory potential was intensified by its entrapment within the schizophyllan-based nanovehicle. Furthermore, the nanovehicles succeeded in enhancing the bioavailability of TLR-9 agonist to its receptor within the endosomes of alveolar macrophages and induced substantial increases in ROS and Th1 inflammatory cytokines, which triggered the generation of an M1 base phenotype.

## 4. Materials and Methods

### 4.1. Materials

Mouse-specific CpG ODN 1826 with sequence 5′-T*C*C*A*T*G*A*C*G*T*T*C*C* T*G*A*C*G*T*T-3′ (* represents a phosphorothioated backbone) was procured from Integrated DNA Technologies (San Diego, CA, USA). Schizophyllan, with a molecular weight of 450 kDa, was procured from Invivogen (San Diego, CA, USA). For fluorescence microscopic studies, CpG ODN 1826 tagged with 6-FAM (derivative of fluorescein, Integrated DNA Technologies, San Diego, CA, USA) with a 5′-end was used. Branched polyethylenimine (PEI) with a molecular weight of 25 KDa, lipopolysaccharide (LPS; E. coli; serotype 055: B5), and 2′, 7′-dichlorodihydrofluorescein diacetate (DCFH-DA) were obtained from Sigma-Aldrich (St. Louis, MO, USA). Dulbecco’s modified eagle medium (DMEM, Hi-media, Mumbai, India), an antibiotic antimycotic solution, and trypan blue 0.4% solution in Dulbecco’s phosphate-buffered saline were from Hi-media, Mumbai, India. Fetal bovine serum (FBS) was procured from Thermo Fisher Scientific (Waltham, MA, USA). N-(3-Triethylammoniumpropyl)-4-(6-(4-(Diethylamino) Phenyl) Hexatrienyl) Pyridinium Dibromide (FM 4-64) was obtained from Invitrogen (Thermo Fisher Scientific, Waltham, MA, USA).

### 4.2. Synthesis of SPG-ODN 1826 Nanovehicles

Complexation between SPG and CpG ODN 1826 was achieved without the use of poly-(A) tails in the ODN as per the protocol described earlier [27]. Initially, SPG was denatured into a single helix by being dissolved in DMSO at a concentration of 1 mg/mL and was then left for ultrasonic degradation for 4 h. The molar N/P ratio between SPG, CpG ODN, and PEI was fixed to 0.1886:10, respectively. After ultrasonic degradation, Tris-HCl and NaCl were added to the reaction mixture at final concentrations of 20 and 50 mM, respectively. CpG ODN was added to the reaction mixture and finally the pH was adjusted to neutral. The final composition of the reaction mixture per mL containing the SPG, PEI, and CpG ODN was 10.6 μM, 93.8 nM, and 2 μM, respectively. The reaction mixture was further incubated overnight at room temperature and then filtered using a 0.2 micron filter before making any measurements.

### 4.3. Characterization of Nanoparticles

#### 4.3.1. Energy Dispersive X-ray (EDX)

We carefully pipetted 50 μL of nanovehicle suspension on a clean borosilicate glass slide and were dried for 1 h under controlled temperature at 50 °C. The glass slide was then scanned using a JEOL 7610F SEM (Tokyo, Japan) at an operating voltage of 25 kV and the spectra were acquired with the aid of EDAX-TEAM software (Version 4.5) considering the sensitivity factor (k-factor) of the different elements.

#### 4.3.2. TEM of SPG-ODN 1826 Nanovehicles

Synthesized nanovehicles were evaluated through TEM for determination of their size and morphology using standard procedures. Then, 20 μL of suspension was placed on the TEM grid and were left overnight. The dried grid was then visualized using a JEOL JEM2100 TEM (JEOL Ltd., Tokyo, Japan).

### 4.4. In Vitro Studies and Cell Culture Conditions

The alveolar murine macrophages (J774A.1) used in the study were obtained from the National Center for Cell Science, Pune, India. J774A.1 cells were maintained in DMEM-high glucose supplemented with 10% FBS and 1% antibiotic-antimycotic solution in a humidified atmosphere with 5% CO_2_ at 37 °C. The cells were stimulated using LPS (1 μg/mL) for at least 4 h and served as the positive control. Flow cytometric analysis was conducted using a BD FACSCalibur flow cytometer (BD Biosciences, San Jose, CA, USA), where at least 10,000 events were analyzed for each experiment.

### 4.5. Evaluation of SPG-ODN 1826 Nanovehicles Induced Cell Cytotoxicity

The synthesized nanovehicles were evaluated for their cytotoxic potential on J774A.1 cells using the standard MTT assay as described previously [46]. During the assay, at least 10^4^ J774A.1 cells were seeded in each well of a 96-well plate and were allowed to adhere in a standard culture environment overnight. LPS-stimulated cells were thereafter treated with either CpG ODN1826 or SPG-ODN 1826 nanovehicles (2 μm) and 96-well plate was further left undisturbed in ambient culture conditions for 24 h. Post-incubation media in each well were decanted and replaced with MTT (5 mg/mL, 100 μL) followed by another incubation of 4 h. Finally, DMSO was supplemented in each well to solubilize the formazan crystals before recording absorbance of SPG-ODN 1826 treated J774A.1 cells at 570 nm. Cell viability percentage was calculated using the following equation

A_T_ × 100/A_C_

where A_T_ is the absorbance of treated J774A.1 cells and A_C_ is the absorbance of LPS-stimulated, non-treated J774A.1 cells.

### 4.6. Determination of Intracellular Localization of SPG-ODN 1826 Nanovehicles

Intracellular localization of SPG-ODN 1826 was analyzed through confocal laser scanning microscopy (CLSM, Zeiss, LSM 780NLO, Oberkochen, Germany) as previously described [27]. In brief, 1.5 × 10^4^ J774A.1 cells were transferred onto a single chamber cell culture slide (SP Lifesciences) and were allowed to adhere. The J774A.1 cells were stimulated using LPS as stated above. Post-stimulation, the cells were exposed to SPG-ODN 1826 nanovehicles for 6 h. Subsequently, the cells were stained using FM 4-64 following the manufacturer’s instructions, and fixed using 4% paraformaldehyde. The slides were finally analyzed using a Carl Zeiss microscope (Zeiss, LSM 780NLO, Oberkochen, Germany).

### 4.7. Assessment of Intracellular ROS Levels

Qualitative assessment of the intracellular ROS levels with SPG-ODN 1826 treated J774A.1 cells was accomplished as described earlier [47]. For the assay, 1 × 10^4^ J774A.1 cells/well were seeded in a 96-well plate and allowed to adhere under optimum culture conditions. Cells were stimulated with LPS and then treated with either CpG ODN1826 or SPG-ODN 1826 nanovehicles for 6 h. Thereafter, media in each well were decanted and replaced with 20 μM of DCFH-DA. The cells were further incubated for 30 min under standard culture conditions, after which they were washed gently and visualized in the FITC channel of a Floid imaging station (Thermo Scientific, Waltham, MA, USA). For quantitatively assessing the level of ROS, the above-stated procedure was used, and the DCF-DA-mediated fluorescence intensity was recorded at an excitation/emission ratio of 485/528 nm using a 96-well black bottom ELISA well through a Synergy H1 Hybrid fluorescent Reader (BioTek, Winooski, VT, USA).

### 4.8. Assessment of Inflammatory Cytokines Levels

The intracellular levels of signature inflammatory cytokines, namely IFN-γ and IL-1β, were estimated using cytometric bead array kits through the standard procedure. Initially, 1 × 10^6^ J774A.1 cells/well were seeded in a 6-well plate and were allowed to adhere overnight. Post-stimulation with LPS, the cells were treated with SPG-ODN 1826 (dilution factor 10) nanovehicles and 2 μM of CpG ODN 1826 and incubated for a specified time (5 h for IFN-γ and 4 h for IL-1β). After incubation, the cells were treated with IFN-γ and IL-1β capture and detection beads following the manufacturer’s protocol. Eventually, the cells were fixed using 4% paraformaldehyde and analyzed for PE-mediated fluorescence through flow cytometry using 575/40 nm excitation and 585/40 nm emission filters.

### 4.9. Statistical Analysis

The data illustrated are representative of three sets of independent experiments with their SEM and each experiment was conducted in triplets. The analysis of results was performed through one-way ANOVA followed by Tukey’s multiple comparison post hoc test using GraphPad Prism (version 5.0). Differences of *p* < 0.05 between the treated groups were considered statistically significant.

## 5. Conclusions

The findings of the current study indicated that the use of the formulated SPG-ODN 1826 nanovehicles has the potential for further in vivo exploration owing to its immunostimulatory properties and because of its efficacy in inducing a prominent M1 or an inflammatory phenotype within the alveolar macrophages, which could pave the way for plausible immunotherapeutic interventions against lung carcinomas.

## Data Availability

The data presented in this study are available on request from the corresponding author.
